# The Swedish version of the multidimensional inventory for religious/spiritual well-being – Part II: Development of a four-field typology

**DOI:** 10.3389/fpsyg.2022.1029101

**Published:** 2022-11-11

**Authors:** Nikita Podolin-Danner, Magdalena Wenzl, Anna Knorr, Jürgen Fuchshuber, Giorgia Silani, Human-Friedrich Unterrainer

**Affiliations:** ^1^Faculty of Psychology, University of Vienna, Vienna, Austria; ^2^Center for Integrative Addiction Research (CIAR), Grüner Kreis Society, Vienna, Austria; ^3^Department of Philosophy, University of Vienna, Vienna, Austria; ^4^Department of Psychiatry and Psychotherapeutic Medicine, Medical University of Graz, Graz, Austria; ^5^Department of Religious Studies, University of Vienna, Vienna, Austria

**Keywords:** cluster analysis, mental health, personality dimensions, religious/spiritual well-being, sense of coherence, Sweden

## Abstract

**Background:**

In the field of mental health, religiosity and spirituality have gained particular attention in recent decades. However, only a few studies to date have investigated the effects of different types of religiosity and spirituality. In association with the recent introduction of a Swedish version of the multidimensional inventory of religious/spiritual well-being (MI-RSWB-S), the present study aimed to identify possible types of Religious/Spiritual Well-Being by using cluster analyses and to examine the extracted groups for differences in the sense of coherence (SOC), the Big Five personality factors, and central aspects of religiosity. Additionally, the study design was intended to further contribute to the validation of the MI-RSWB-S.

**Methods:**

Based on a convenience sample of Swedish students (*N* = 1,011), initially obtained for the development of the MI-RSWB-S, the study included the MI-RSWB-S, the 13-items sense of coherence scale, the 10-item personality inventory, and the centrality of religiosity scale. For the statistical analysis, cluster analyses and one-way analyses of variance (ANOVAs) were conducted.

**Results:**

The cluster analyses yielded the following four groups: Religiosity and spirituality high (*n* = 124), religiously oriented (*n* = 200), spiritually oriented (*n* = 149), and religiosity and spirituality low (*n* = 538). The groups differed in most aspects of well-being, in the personality dimensions agreeableness and openness to experience, as well as in central aspects of religiosity. In contrast, no differences were found for SOC, extraversion, conscientiousness, and emotional instability.

**Conclusion:**

Our results suggest that different types of religious/spiritual well-being are associated with mental health and personality dimensions in substantially different ways, thus offering an interesting potential for future research.

## Introduction

The study of the relationship between religiosity and spirituality on the one hand and (mental) health on the other is the most extensive element of research in the field of psychology of religion (Saroglou, 2021). Yet, the results are overall mixed and complex: While religiosity and spirituality are frequently associated with positive mental health (e.g., lower rates of depression, anxiety, and substance use as well as higher subjective well-being and sense of purpose), they are also associated with certain negative outcomes (e.g., cognitive rigidity, delayed professional consultation, faith related struggles, obsessionality, and sexual repression; for a review, see Koenig et al., 2012; Saroglou, 2021). To explain the contradictory findings, several authors have argued that the results strongly depend on the definition of these constructs (e.g., Hall et al., 2008; Hodapp and Zwingmann, 2019). The great research interest in the interface between mental health and the psychology of religion therefore underlines the need for specialized instruments that capture the complexity of immanent as well as transcendent aspects of mental health.

The multidimensional inventory of religious/spiritual well-being (MI-RSWB; Unterrainer et al., 2010a) was developed within a European religious-sociological background to capture self-reported well-being in a comprehensive way by combining an immanent area as well as a transcendent area of well-being. The former compromises the variables Forgiveness (FO), Hope Immanent (HI), representing optimistic attitudes towards one’s future, and Experience of Sense and Meaning (SM). Although these variables do not necessarily have a transcendent connotation, they are often the subject of research in the psychology of religion and are frequently associated with religious and spiritual facets in a positive way (e.g., Emmons, 2005; Ciarrocchi et al., 2008; Fincham et al., 2020). The transcendent area consists of General Religiosity (GR), Connectedness (CO), and Hope Transcendent (HT). GR is hereby defined as a traditional and institutionalized religious faith, whereas CO is described as an undogmatic form of belief and the feeling of being immersed in something bigger than oneself, compromising the main elements of a common definition for spirituality (see Saroglou, 2021). Additionally, HT extracts ideas of a life beyond death and focuses on this belief in particular.

The explicit distinction between GR and CO, is meant to reflect the observation that modern conceptions of religiosity and spirituality diverge in research as well as in society (Saucier and Skrzypińska, 2006). For example, many people who feel connected to everything are inclined to see themselves as spiritual but not necessarily religious (Diebels and Leary, 2019). This phenomenon is particularly true in modern secular societies, where religiosity and spirituality are interconnected rather than interchangeable, as is the case in traditional communities (Saroglou, 2021). Consistently, a number of studies using the MI-RSWB in a European context have shown that GR and CO are each frequently associated with different facets of mental health as well as different dimensions of personality, while being constantly interrelated (Unterrainer et al., 2010b, 2012, 2013, 2014; Kämmerle et al., 2014; Hiebler-Ragger et al., 2018). Considering the well documented distinct, though in some respects intertwined, nature of religiosity and spirituality, it can be argued that their interaction could be captured in a typology. Accordingly, Unterrainer et al. (2011) identified four types of Religious/Spiritual Well-Being in a sample of the Austrian general population using a cluster analytical approach that focused on the dimensions of the MI-RSWB. Subsequently, they concluded that different types of Religious/Spiritual Well-Being are associated with mental health and personality in different ways. However, research on different types of religiosity and spirituality seems to remain marginal and there is little literature focusing on their interaction in general (Kao et al., 2020).

In a recent project, the MI-RSWB was translated into Swedish (MI-RSWB-S; Wenzl et al., 2021), shifting the focus to one of the most secular countries in the world (Esmer and Pettersson, 2007). In the course of the validation process, the instrument was thoroughly examined for its statistical properties, resulting in a minor adaptation of its factorial structure (Wenzl et al., 2021). Briefly, the division into a transcendent and existential area of well-being was rejected and a new general factor was introduced to the revised MI-RSWB (MI-RSWB-R), that is, the revised religious/spiritual well-being (RSWB-R). The general factor comprises the dimensions GR and CO, which, according to theoretical considerations, can also be named religious well-being (RWB) and spiritual well-being (SWB). As in previous studies with a European background, Wenzl et al. (2021) subsequently found that GR (RWB) and CO (SWB) had similarly different correlates in Swedish participants, while still being strongly interrelated (*r* = 0.66, *p* < 0.01). Along with the recent advances in the development of the MI-RSWB and the theoretical as well as empirical findings of Unterrainer et al. (2011), these results provide a suitable basis for introducing a novel typology of Religious/Spiritual Well-Being.

## Study aims

The present study intends to (a) complement the general lack of research on different types of religiosity and spirituality and to (b) additionally contribute to the validation of the MI-RSWB-S (Wenzl et al., 2021). To this end, two consecutive steps were conceived, following the approach of Unterrainer et al. (2011): First, cluster analyses are conducted to identify possible types of Religious/Spiritual Well-Being in a convenience sample of Swedish students. The participants were originally recruited for the development of the Swedish version of the MI-RSWB (for further details see Wenzl et al., 2021). Second, the extracted clusters are examined for differences in the well-being dimensions of the MI-RSWB, the sense of coherence (SOC), the Big Five personality factors, and key aspects of religiosity.

## Materials and methods

### Participants and procedure

Swedish students were approached by announcements on social media (Facebook and Instagram). Before being given access to the online survey, participants were directed to download a PDF providing detailed information about the study. After indicating their consent, participants received access to the online survey. The survey and all the questionnaires included were presented in Swedish and data were collected by using the survey program SoSci Survey. Between March 8, 2021 and April 5, 2021, 1,051 people completed the entire survey, qualifying for consideration of the following inclusion criteria: (1) Swedish citizenship, (2) Swedish fluency, (3) student enrolment at a Swedish university or university college, (4) age between 18 and 40 years, and (5) a minimum of 4 min to complete the survey (time criterion). After excluding participants who did not match these criteria, the final sample consisted of 1,011 participants. Table 1 summarizes selected sociodemographic characteristics of the study sample (for a comprehensive presentation of the sample and procedure, see Wenzl et al., 2021). Ethical approval was granted by the Ethics Committee of the University of Vienna.

**Table 1:** Selected sociodemographic characteristics.

Religious affiliation	*n* (%)	Gender	*n* (%)
Christianity (Church of Sweden)	467 (46.2)	Female	747 (73.9)
Christianity (free church)	102 (10.0)	Male	252 (24.9)
Christianity (Eastern Orthodox)	33 (3.3)	Other	12 (1.2)
Christianity (Roman Catholic)	30 (3.0)		
Christianity (other)	3 (0.3)	Age	
Islam (Sunni)	59 (5.8)	18–19	63 (6.2)
Islam (Shia)	10 (1.0)	20–24	525 (51.9)
Islam (other)	2 (0.2)	25–29	263 (26.0)
Judaism	13 (1.3)	30–34	100 (9.9)
Buddhism	13 (1.3)	35–40	60 (5.9)
Hinduism	2 (0.2)		
I have never been a part of a religious community	166 (16.4)		
I have left a religious community	156 (15.4)		
Other	10 (1.0)		

*N* = 1011.

### Measurements

#### Multidimensional inventory for religious/spiritual well-being

The MI-RSWB (Unterrainer et al., 2010a) consists of 48 items which are equally distributed across six subscales. According to the new structure of the Swedish version of the instrument (MI-RSWB-S), the subscales RWB (e.g., “My faith gives me a feeling of security.”) and SWB (e.g., “There are people with whom I feel a supernatural connection.”) account for the joint factor Religious/Spiritual Well-Being Revised (RSWB-R), on the one side. On the other side, the dimensions FO (e.g., “There are things which I cannot forgive.” [reversed]), HI (e.g., “I think that things will improve in the future.”), HT (e.g., “I would do anything to prolong my life.” [reversed]), and SM (e.g., “I have experienced deep affection.”) separately represent positive indicators of well-being. The items are rated by using a six-point Likert scale, ranging from (1) *totally disagree* to (6) *totally agree*. For the MI-RSWB-S, Cronbach’s α was between 0.67 (SM) and 0.97 (GR) for the subscales and 0.94 for the joint factor RSWB-R. Accordingly, the reliability of the MI-RSWB was generally shown to be consistent across different translations (e.g., Unterrainer et al., 2010a, 2012; Stefa-Missagli et al., 2014; Berger et al., 2016; Malinovic et al., 2016; Agarkov et al., 2018), with the exception of the Farsi adaptation, where Cronbach’s α for HT and SM was considerably lower (see Dadfar et al., 2019; Kazemzadeh Atoofi et al., 2019).

#### 10-item personality inventory

The TIPI (Gosling et al., 2003) is a personality questionnaire based on the Five-Factor Model (Costa and McCrae, 1992). Generally known as the Big Five, the TIPI comprises five universal personality dimensions: extraversion, agreeableness, conscientiousness, openness to experience, and emotional stability, which is the opposite of the original dimension neuroticism, or emotional instability. For the purpose of this study, however, the latter scale was reversed to assess the original dimension Emotional Instability. The TIPI was conceptualized to measure each of these dimensions by two items (e.g., openness to experience: “I see myself as open to new experiences, complex.”). Each item is accompanied by a Likert scale ranging from (1) *disagree strongly* to (7) *agree strongly*. According to the guidelines of George and Mallery (2016), Extraversion (α = 0.75) and Emotional Instability (α = 0.70) reached an acceptable level of internal consistency, while Conscientiousness (α = 0.57) is considered as poor. Agreeableness (α = 0.24) and Openness to Experience (α = 0.40), however, showed unacceptable levels of Cronbach’s α, challenging earlier reports (Gosling et al., 2003). The Swedish version of the TIPI first appeared in Lundell (2014).

#### Sense of coherence scale

The SOC-13 Scale (short version of the original SOC-29) measures the SOC, which is a crucial element of Antonovsky (1987) model of Salutogenesis. SOC can be understood as a global orientation that enables an individual to cope with stress and maintain health through a pervasive–enduring though dynamic–feeling of confidence. SOC comprises three components, that are: (a) a thorough understanding of the environment and inner experiences (Comprehensibility), (b) the perceived availability of means and resources to meet their demands (Manageability), and (c) finding Meaningfulness in this engagement (Antonovsky, 1987, 1991a). SOC has been reported to be strongly related to perceived mental health and is thus considered as a crucial indicator of well-being (Eriksson and Lindström, 2007). A seven-point Likert scale with varying verbal response anchors is provided for each item of the SOC-13 Scale (e.g., Comprehensibility: “Do you have very mixed-up feelings and ideas?”). For the purpose of our study, only the total score was considered for analysis. The internal consistency for the Swedish version of the SOC-13 scale (Cronbach’s α) was 0.83, which is consistent with earlier reports (e.g., Larsson and Kallenberg, 1999; Räty et al., 2003). The Swedish translation of the SOC-13 (KASAM-13) was first published in the Swedish book “Hälsans mysterium” (Antonovsky, 1991b).

#### Centrality of religiosity scale

The centrality of religiosity scale (CRS) captures the centrality, significance, and salience of religious matters in a person’s life (Huber and Huber, 2012). Within the CRS, five dimensions of religiosity are measured: public practice, private practice, religious experience, ideology, and intellect. All dimensions put together portray a global picture of an individual’s religious life. The 5-item version of the scale (CSR-5) provides one item (e.g., Intellect: “How often do you think about religious issues?”) for each dimension. The items are rated on a five-point (religious experience, ideology, and intellect), six-point (Public Practice), or eight-point Likert scale (Private Practice), accompanied by different verbal response anchors. In retrospect, the answers on the six-and eight-point scales are converted into values between 1 and 5, as specified in the instructions. In terms of internal consistency, the overall scale achieved a Cronbach’s α of 0.92, outperforming previous results (Huber and Huber, 2012). The Swedish version of the CRS-5 (CRS-5 SWE; Sjöborg, 2014) was used for the survey. Unlike the original version, the CRS-5 SWE additionally provides a “do not know” response option besides the described answer scales.

### Study design and data analysis

Given the research goals and the composition of the sample, the study comprises explorative and cross-sectional elements of cluster analyses and between-subjects examinations. A cluster analysis was conducted combining the variables RWB and SWB. In order to find an appropriate typology of religious and spiritual well-being, two types of clustering – a hierarchical cluster analysis using WARD-method combined with squared euclidean distance and a two-step cluster analysis – were performed and compared. In a second step, the extracted clusters, or groups, functioned as levels of the independent variable “Types of Religious/Spiritual Well-Being.” Accordingly, these were explored for differences in 14 dependent variables, namely the subscales of the MI-RSWB-S (RWB, SWB, HT, FO, HI, and SM) and the joint factor RSWB-R, the five personality dimensions (Extraversion, Agreeableness, Conscientiousness, Emotional Instability, and Openness to Experience), SOC, and CRS. For the statistical analysis, multiple Univariate Analyses of Variance (one-way ANOVAs) were conducted, which is considered an adequate approach for the present research question (Huberty and Morris, 1989). The *p*-values were adjusted by using Bonferroni-Holm correction. Lastly, Tukey and Games-Howell tests were performed for post-hoc intergroup comparisons. All results were interpreted based on a two-sided alpha-level with *p* = 0.05.

## Results

### Cluster analysis

First, a hierarchical cluster analysis was conducted on the two variables RWB and SWB, followed by an examination of the dendrogram for possible outcomes. With respect to theoretical considerations and the inspection of the dendrogram, a four-cluster-solution was aspired. As data order has been reported to potentially affect the analysis (Van der Kloot et al., 2005), at least 10 differently ordered samples were clustered by following the exact same methods. The solutions, however, did not differ considerably from each other. Furthermore, outliers are known to strongly influence the clustering process (Everitt et al., 2010). Two outliers were found for SWB. After exclusion, a new cluster analysis was exploratively performed on the reduced sample and characteristics of the clusters (means, medians, and standard deviations) were compared to those of the original cluster solution. The observed differences were small and considered insignificant regarding the interpretation of the clusters. Therefore, the outliers were reintroduced into the further process. Finally, a two-step cluster analysis with a predefined four-cluster solution was conducted. With slightly different results, the new variant best matched our theoretical conceptions of a typology of religious and spiritual well-being. The computed clusters were named according to their unique combination of the variables RWB and SWB: Religiosity and Spirituality high (RS-h), Religiously Oriented (RO), Spiritually Oriented (SO) and Religiosity and Spirituality low (RS-l). The differentiation between “high,” “mid-high,” “mid-low,” and “low” expressions of RWB and SWB was based on the relative comparison between the centroids of each cluster. The main purpose of this typology was to facilitate the discrimination between the investigated groups. In Table 2, a profile (group size, centroids, and typology) for each cluster of the two-step cluster solution is provided.

**Table 2:** Profiles for the four-cluster solution of the two-step cluster analysis and a typology of religious and spiritual well-being.

		RWB		SWB		Typology	
Cluster	*N*	*M*	*SD*	*M*	*SD*	*RWB*	*SWB*
RS-h	124	5.28	0.61	4.64	0.62	High	High
RO	200	4.82	0.80	3.07	0.54	High	Mid-low
SO	149	2.24	0.84	3.86	0.63	Low	Mid-high
RS-l	538	1.30	0.41	1.97	0.52	Low	Low

RWB, religious well-being; SWB, spiritual well-being; RS-h, religiosity and spirituality high; RO, religiously oriented; SO, spiritually oriented; RS-l, religiosity and spirituality.

### Univariate analyses of variance

In terms of validation, one-way ANOVAs were scheduled to investigate differences between the clusters in all 14 variables (RSWB-R, RWB, SWB, FO, HT, HI, SM, extraversion, agreeableness, conscientiousness, emotional instability, openness to Experience, SOC, and CRS). 13 variables were analyzed including the total study sample of 1,011 participants. Regarding the CRS-5, however, 7 participants had to be excluded from statistical analysis within this variable (*n* = 1004), due to considerations related to the “do not know”-response option.

First, variables were checked for preconditions of the one-way ANOVAs. By studying box plots, 14 extreme outliers were found for cluster RS-l to score higher on the variable RWB and two extreme outliers in group RO scored lower on HI. Several other outliers were considered insignificant. Finally, Levene test revealed no homogeneity of variances for CRS, RSWB, and all dimensions of the MI-RSWB but FO and HT (*p* < 0.5). To control for the influence of the listed violations of preconditions, outputs of Welch’s ANOVAs (robust with respect to violations of variance homogeneities) and Kruskal-Wallis-H tests (robust with respect to violations to outliers) were compared to the one-way ANOVAs. Since they yielded no differences in interpretation of significances, the analysis proceeded as usual. Table 3 summarizes descriptive and Table 4 interferential statistics (one-way ANOVAs and post-hoc tests) of cluster differences regarding the 14 dependent variables.

**Table 3:** Descriptive statistics for the MI-RSWB (subscales and joint factor), TIPI, SOC-13 scale, and CRS-5.

	RS-h			RO			SO			RS-l		
	*N*	*M*	*SD*	*N*	*M*	*SD*	*N*	*M*	*SD*	*N*	*M*	*SD*
MI-RSWB (MI-RSWB-S)												
Religious/spiritual well-being revised	124	4.96	0.40	200	3.95	0.54	149	3.05	0.59	538	1.64	0.37
Religious well-being	124	5.28	0.61	200	4.82	0.80	149	2.24	0.84	538	1.30	0.41
Spiritual well-being	124	4.64	0.62	200	3.07	0.54	149	3.86	0.63	538	1.97	0.52
Hope transcendent	124	4.30	0.98	200	4.27	1.00	149	4.14	0.91	538	4.30	0.92
Forgiveness	124	4.89	0.99	200	4.79	0.99	149	4.08	1.03	538	4.07	1.01
Hope immanent	124	4.67	0.64	200	4.45	0.80	149	4.37	0.83	538	4.19	0.86
Experience of sense and meaning	124	5.22	0.56	200	4.70	0.71	149	4.73	0.67	538	4.21	0.73
Tipi												
Extraversion	124	4.98	1.37	200	4.77	1.43	149	4.92	1.31	538	4.70	1.40
Agreeableness	124	5.63	0.87	200	5.42	0.97	149	5.46	0.89	538	5.26	0.96
Conscientiousness	124	5.16	1.12	200	5.01	1.21	149	4.81	1.27	538	4.92	1.24
Emotional instability	124	3.28	1.32	200	3.39	1.34	149	3.51	1.38	538	3.48	1.46
Openness to experience	124	5.77	0.95	200	5.31	1.04	149	5.58	0.97	538	5.20	1.04
SOC-13 scale (KASAM-13)												
Sense of coherence	124	4.44	0.80	200	4.35	0.88	149	4.25	0.94	538	4.33	0.88
CRS-5 (CRS-5 SWE)												
Centrality of religiosity scale	123	4.04	0.67	198	3.81	0.79	148	2.32	0.63	535	1.55	0.43

RS-h, religiosity and spirituality high; RO, religiously oriented; SO, spiritually oriented; RS-l, religiosity and spirituality low; MI-RSWB (MI-RSWB-S), multidimensional inventory for religious/spiritual well-being (Swedish Version); TIPI, 10-item personality inventory; SOC-13 Scale (KASAM-13), 13-item version of the sense of coherence scale (Swedish Version); CRS-5 (CRS-5 SWE), 5-item version of the centrality of religiosity scale (Swedish Version).

**Table 4:** ANOVAs and intergroup comparisons for the MI-RSWB (subscales and joint factor), TIPI, SOC-13 scale, and CRS-5.

		ANOVA			Intergroup comparisons	
	*N*	*F*	*p*	η^2^	Results	*p*
MI-RSWB (MI-RSWB-S)						
Religious/spiritual well-being revised^1^	1011	2590.68	<0.001	0.885	RS-h > RO > SO > RS-l	<0.001
Religious well-being^1^	1011	2539.31	<0.001	0.883	RS-h > RO > SO > RS-l	<0.001
Spiritual well-being^1^	1011	1061.83	<0.001	0.760	RS-h > SO > RO > RS-l	<0.001
Hope transcendent^1^	1011	1.15	0.98	-		
Forgiveness^1^	1011	41.18	<0.001	0.109	RS-h, RO > SO, RS-l	<0.001
Hope immanent^1^	1011	13.50	<0.001	0.039	RS-h > SO, RS-l / RO > RS-l	<0.05 / 0.001
Experience of sense and meaning^1^	1011	84.50	<0.001	0.201	RS-h > SO, RO > RS-l	<0.001
TIPI						
Extraversion^1^	1011	2.03	0.46	-		
Agreeableness^1^	1011	6.08	<0.001	0.035	RS-h > RS-l	0.001
Conscientiousness^1^		2.14	0.46	-		
Emotional instability^1^	1011	0.83	0.98	-		
Openness to experience^1^	1011	13.32	<0.001	0.062	RS-h > RS-l, RO / SO > RS-l	<0.001 / 0.001
SOC-13 scale (KASAM-13)						
Sense of coherence^1^	1011	1.09	0.98	-		
CRS-5 (CRS-5 SWE)						
Centrality of religiosity scale^2^	1004	1106.80	<0.001	0.769	RS-h > RO > SO > RS-l	<0.05

1 Results for *F*(3,1007).

2 Results for *F*(3,1000). RS-h, religiosity and spirituality high; RO, religiously oriented; SO, spiritually oriented; RS-l, religiosity and spirituality low; MI-RSWB (MI-RSWB-S), multidimensional Inventory for religious/spiritual well-being (Swedish Version); TIPI, 10-item personality inventory; SOC-13 Scale (KASAM-13), 13-item version of the sense of coherence scale (Swedish Version); CRS-5 (CRS-5 SWE), 5-item version of the centrality of religiosity scale (Swedish Version). *p*-values are Bonferroni-Holm corrected.

ANOVAs revealed significant cluster differences in RSWB-R and all subscales of the MI-RSWB-S (*p* < 0.001), except for HT (*p* = 0.98; for precise statistics, see Table 4). Post-hoc tests indicated that RS-h scored highest on RSWB-R and RWB, followed by RO, SO, and RS-l (*p* < 0.001 for all comparisons). For SWB, RO and SO switched positions (*p* < 0.001). Regarding FO, the groups RS-h and RO (both high in religiosity) were superior (*p* < 0.001) to RS-l and SO (both low in religiosity). In terms of HI, RS-h scored highest (*p* < 0.05), whereas RO outperformed RS-l (*p* = 0.001), but did not differ significantly from SO (*p* = 0.77). No differences were found between RS-l and SO (*p* = 0.12). Lastly, RS-h obtained the highest score (*p* < 0.001) on SM, followed by SO and RO, which scored equally high (*p* = 0.97), and finally RS-l, showing the lowest expressions of SM (*p* < 0.001).

In terms of Agreeableness, RS-h scored higher than RS-l (*p* = 0.001), whereas no differences were found between the other groups (*p* > 0.05). Openness to Experience was higher for RS-h compared to RO and RS-l (*p* < 0.001). Additionally, the latter group scored lower than SO (*p* = 0.001), while RO and SO did not differ significantly (*p* = 0.07). Regarding the other personality dimensions, ANOVAs did not indicate significance for Extraversion (*p* = 0.43), Conscientiousness (*p* = 0.46), or Emotional Instability (*p* = 0.98). Similarly, no differences were found for SOC (*p* = 0.98). However, the clusters differed significantly in CRS, revealing the following order: RS-h > RO > SO > RS-l (at least *p* < 0.05).

## Discussion

Based on the Swedish version of the MI-RSWB (MI-RSWB-S), the present study intended to a introduce a typology of religious/spiritual well-being that is based on a Swedish study sample as well as on the revised factorial structure of the MI-RSWB-S and b) relate the resulting types to different facets of well-being, the SOC, the Big-Five personality dimensions, and to central aspects of religiosity. To determine different types of Religious/Spiritual Well-Being, a hierarchical and a consecutive two-step cluster analysis were conducted, focusing on the RWB and SWB dimensions of the MI-RSWB-S. The final cluster solution yielded four groups of participants, that are, RS-h (*n* = 124), RO (*n* = 200), SO (*n* = 149), and RS-l (*n* = 538).

The SO and RS-l clusters make up more than 60% of participants that are accordingly low in RWB. This finding is consistent with the observation of a nationally representative survey in which 60% of respondents reported not to believe in God (Pew Research Center, 2018). Accordingly, the present study sample reflects the high level of secularism generally documented in the Swedish population (Esmer and Pettersson, 2007). Moreover, the relatively comparable distribution across RS-h, RO, and SO reflects the notion that religiosity and spirituality are understood as distinct rather than interchangeable in secular societies (Saroglou, 2021). When comparing the clusters in terms of subjective well-being, personality dimensions, and CRS, significant differences with very large effect sizes (*p* < 0.001, at least η^2^ = 0.760) were found for RWB, SWB, and the joint factor RSWB-R. Because the cluster analysis was centered around the two variables RWB and SWB, the fact that the clusters differed clearly from each other validates the accuracy of the accepted cluster solution (see Table 2).

With respect to FO, HI, and SM, our findings are in line with prior studies showing positive correlates with RWB (referred to as GR) and SWB (referred to as CO) across different countries and translations of the MI-RSWB (Unterrainer et al., 2012; Berger et al., 2016; Malinovic et al., 2016; Agarkov et al., 2018). Apart from purely correlational and cross-sectional studies, there is general evidence that certain religious and spiritual aspects might predict forgiveness, hope and optimism, as well as sense of meaning and purpose in a causal way (Ciarrocchi et al., 2008; Van Tilburg et al., 2019; Fincham et al., 2020). Moreover, they predict subjective well-being beyond mediational effects of the Big-Five personality dimensions, which were found to explain a great part of the interindividual differences in religiosity and spirituality in general (Ciarrocchi et al., 2008). Finally, differences in HT were not significant. In accordance with correlative findings (Unterrainer et al., 2012; Berger et al., 2016; Malinovic et al., 2016; Agarkov et al., 2018; Wenzl et al., 2021), our results therefore support the notion that people can equally rely on transcendent as well as on existential beliefs when confronted with the threat of death (Wenzl et al., 2021).

In terms of personality dimensions, Extraversion is usually not considered a relevant associate of religiosity and spirituality (e.g., Yonker et al., 2012). However, spiritual individuals, as well as individuals who adopt a particular form of religiosity (e.g., an open/liberal or positive-coping-related religiosity; see Saroglou, 2002), appear to score relatively high on Extraversion. It is therefore surprising that no differences in Extraversion were found between the present groups. Similarly, Openness to Experience is generally described as more typical for (modern) spiritual people than for religious individuals, whereas Agreeableness and Conscientiousness are characteristic for both (Saroglou, 2021). Accordingly, the varying levels of Agreeableness and Openness to Experience between certain clusters seem to point to the same conclusion (see Table 4). However, no differences were found for Conscientiousness. Lastly, Emotional Instability (or Neuroticism) has almost consistently been unrelated to different operationalizations of religiosity and spirituality (e.g., Yonker et al., 2012; Hiebler-Ragger et al., 2018), which is reflected by the present findings.

No significant differences were reported for SOC. This result is in line with Wenzl et al. (2021), where no association with RWB or SWB was found. However, according to Antonovsky (1987), religiosity and spirituality are assumed to be positively related to SOC or even to enhance it. This notion is partly supported by empirical findings showing a positive relationship between SOC and GR (Unterrainer et al., 2010a; Berger et al., 2016). The contradictory findings could be caused by differing populations or by other confounding variables that might be revealed in future studies. Finally, all groups differed remarkably from one another regarding CRS (*p* < 0.001, η^2^ = 0.762). This result is not surprising, but has a valuable implication, as it additionally demonstrates the practical accuracy of the accepted cluster solution in terms of precisely distinguishing participants with different expressions of religiosity (and spirituality).

Overall, our results clearly suggest that different types of Religious/Spiritual Well-Being are associated in different ways with different aspects of well-being and different personality dimensions, thus supporting the conclusion of Unterrainer et al. (2011). On the one hand, however, it is difficult to say whether the differences between types of Religious/Spiritual Well-Being are of an interactive nature or whether they can be fully explained by the individual correlations of RWB and SWB (see Wenzl et al., 2021). In other words, do our results indicate a (partially) interactive relationship between RWB and SWB or is it purely summative? To answer this question, an important next step will be to analyze if there are moderating or even mediating effects between RWB and SWB or, more generally, between religiosity and spirituality. Regardless of whether the effects are interactive or summative, we conclude that looking at typologies is an elegant way to represent and interpret the combined effects of religiosity and spirituality, as opposed to considering individual correlations alone. Finally, the present study contributed to the validation of the revised MI-RSWB by showing that the instrument can be used to investigate diverse questions in the field of the psychology of religion or mental health and is compatible with different statistical designs.

## Limitations

Several limitations of the study should be noted. First, the current results are not meant to be generalized to the Swedish population, as inferences were drawn on the basis of a convenience sample of students. Secondly, due to the cross-sectional design of the study, the conducted analyses do not allow for a causal interpretation of the results. However, there is evidence that certain aspects of religiosity and spirituality may indeed function as causal predictors of well-being, while at the same time, some other components, such as negative religious coping and spiritual struggles, can lead to impaired mental health (Ciarrocchi et al., 2008). Finally, the TIPI subscales Agreeableness, Conscientiousness, and Openness to Experience demonstrated remarkably low levels of internal consistency. Even though the subscales have previously demonstrated acceptable test–retest reliabilities (Gosling et al., 2003), the results still need to be interpreted with caution. To address some of these limitations, future studies could use more comprehensive instruments to measure personality and focus on nationally representative study samples. In addition, we see the need to consider the influence of competing definitions of mental health, religiosity and spirituality, as well as the impact of different contexts and cultures. In this regard, particularly clinical populations should be addressed, as different types of Religious/Spiritual Well-Being may provide valuable implications for the work in the clinical field.

## Conclusion and future directions

By taking into account the different, albeit interrelated, nature of religious and spiritual beliefs, we identified four types of Religious/Spiritual Well-Being in a Swedish sample using cluster analyses. By subsequently comparing the four clusters, we were able to provide considerable evidence for their different associations with mental health and personality. To study transcendent beliefs in (post-)modern societies and to understand their role in mental health and beyond, we therefore encourage future research to focus more on the interaction and types of religiosity and spirituality than on their individual properties alone.

## Data availability statement

The raw data supporting the conclusions of this article will be made available by the authors, without undue reservation.

## Ethics statement

The studies involving human participants were reviewed and approved by University of Vienna. The patients/participants provided their written informed consent to participate in this study.

## Author contributions

NP-D and H-FU conceptualized the study. MW and JF acquired the data. NP-D performed all statistical analyses and drafted the manuscript. H-FU, JF, MW, AK, and GS revised and proofread the manuscript. All authors critically discussed the results and contributed to the article and approved the submitted version.

## Conflict of interest

The authors declare that the research was conducted in the absence of any commercial or financial relationships that could be construed as a potential conflict of interest.

## Publisher’s note

All claims expressed in this article are solely those of the authors and do not necessarily represent those of their affiliated organizations, or those of the publisher, the editors and the reviewers. Any product that may be evaluated in this article, or claim that may be made by its manufacturer, is not guaranteed or endorsed by the publisher.

## References

[ref1] Agarkov V. Alexandrov Y. Bronfman S. Chernenko A. Kapfhammer H. Unterrainer H.-F. (2018). A Russian adaptation of the multidimensional inventory for religious/spiritual well-being. Arch. Psychol. Relig. 40, 104–115. doi: 10.1163/15736121-12341347

[ref2] Antonovsky A. (1987). Unraveling the mystery of health: how people manage stress and stay well. San Francisco, CA: Jossey-Bass.

[ref3] Antonovsky A. (1991a). *Hälsans Mysterium [Health’s Mystery]*. Natur och kultur.

[ref4] Antonovsky A. (1991b). “The structural sources of salutogenic strengths,” in Personality and Stress: Individual Differences in the Stress Process. eds. Cooper C. L. Payne R. (John Wiley & Sons), 67–104.

[ref6] Berger D. Fink A. Perez Gomez M. M. Lewis A. Unterrainer H.-F. (2016). The validation of a Spanish version of the multidimensional inventory of religious/spiritual well-being in Mexican college students. Span. J. Psychol. 19, E3–E11. doi: 10.1017/sjp.2016.9, PMID: 26887859

[ref7] Ciarrocchi J. W. Dy-Liacco G. S. Deneke E. (2008). Gods or rituals? Relational faith, spiritual discontent, and religious practices as predictors of hope and optimism. J. Posit. Psychol. 3, 120–136. doi: 10.1080/17439760701760666

[ref8] Costa P. T. McCrae R. R. (1992). Normal personality assessment in clinical practice: the NEO personality inventory. Psychol. Assess. 4, 5–13. doi: 10.1037/1040-3590.4.1.5

[ref9] Dadfar M. Lester D. Turan Y. Beshai J. A. Unterrainer H.-F. (2019). Validation of the multidimensional inventory for religious spiritual well-being with Iranian samples. Mental Health Rel. Cult. 22, 591–601. doi: 10.1080/13674676.2019.1628194

[ref10] Diebels K. J. Leary M. R. (2019). The psychological implications of believing that everything is one. J. Posit. Psychol. 14, 463–473. doi: 10.1080/17439760.2018.1484939

[ref11] Emmons R. A. (2005). Striving for the sacred: personal goals, life meaning, and religion. J. Soc. Issues 61, 731–745. doi: 10.1111/j.1540-4560.2005.00429.x

[ref12] Eriksson M. Lindström B. (2007). Antonovsky’s sense of coherence scale and its relation with quality of life: a systematic review. J. Epidemiol. Com. Health 61, 938–944. doi: 10.1136/jech.2006.056028, PMID: 17933950 PMC2465600

[ref13] Esmer Y. R. Pettersson T. (eds.) (2007). Measuring and Mapping Cultures: 25 Years of Comparative Value Surveys. Leiden: Brill.

[ref14] Everitt B. S. Landau S. Leese M. Stahl D. (2010). Cluster Analysis. 5th Edn New Jersey: Wiley.

[ref15] Fincham F. D. May R. W. Carlos Chavez F. L. (2020). Does being religious lead to greater self-forgiveness? J. Posit. Psychol. 15, 400–406. doi: 10.1080/17439760.2019.1615109

[ref16] George D. Mallery P. (2016). IBM SPSS Statistics 23 Step by Step: A Simple Guide and Reference. 14th Edn New York, NY: Routledge.

[ref17] Gosling S. D. Rentfrow P. J. Swann W. B. (2003). A very brief measure of the big-five personality domains. J. Res. Pers. 37, 504–528. doi: 10.1016/S0092-6566(03)00046-1

[ref18] Hall D. Meador K. Koenig H. (2008). Measuring religiousness in health research: review and critique. J. Relig. Health 47, 134–163. doi: 10.1007/s10943-008-9165-2, PMID: 19105008 PMC8823950

[ref19] Hiebler-Ragger M. Fuchshuber J. Dröscher H. Vajda C. Fink A. Unterrainer H.-F. (2018). Personality influences the relationship between primary emotions and religious/spiritual well-being. Front. Psychol. 9:370. doi: 10.3389/fpsyg.2018.00370, PMID: 29615950 PMC5868126

[ref20] Hodapp B. Zwingmann C. (2019). Religiosity/spirituality and mental health: A meta-analysis of studies from the German-speaking area. J. Relig. Health 58, 1970–1998. doi: 10.1007/s10943-019-00759-0, PMID: 30632002

[ref21] Huber S. Huber O. W. (2012). The centrality of religiosity scale (CRS). Religions 3, 710–724. doi: 10.3390/rel3030710

[ref22] Huberty C. J. Morris J. D. (1989). Multivariate analysis versus multiple univariate analyses. Psychol. Bull. 105, 302–308. doi: 10.1037/0033-2909.105.2.302

[ref23] Kämmerle U. H.-F. Dahmen-Wassenberg P. Fink A. Kapfhammer H.-P. (2014). Dimensions of religious/spiritual well-being and the dark triad of personality. Psychopathology 47, 297–302. doi: 10.1159/000358563, PMID: 24852674

[ref24] Kao L. Peteet J. Cook C. (2020). Spirituality and mental health. J. Study Spiritual. 10, 42–54. doi: 10.1080/20440243.2020.1726048

[ref5] Kazemzadeh Atoofi M. Dadfar M. Turan Y. Behnam L. (2019). Multidimensional inventory for religious spiritual well-being (MI RSWB 48): validation with Iranian psychiatric outpatients. Mental Health Rel. Cult. 22, 1011–1020. doi: 10.1080/13674676.2019

[ref25] Koenig H. G. King D. E. Carson V. B. (2012). Handbook of Religion and Health (2nd Edition). New York, NY: Oxford University Press.

[ref26] Larsson G. Kallenberg K. (1999). Dimensional analysis of sense of coherence using structural equation modelling. Eur. J. Personal. 13, 51–61. doi: 10.1002/(SICI)1099-0984(199901/02)13:1<51::AID-PER321>3.0.CO;2-P

[ref27] Lundell E. (2014). Ten-item personality inventory-(TIPI) – Swedish translation. http://gosling.psy.utexas.edu/wp-content/uploads/2014/09/TIPISwedishtranslation.doc (Accessed September 5, 2021).

[ref28] Malinovic A. Fink A. Lewis A. Unterrainer H.-F. (2016). Dimensions of religious/spiritual well-being in relation to personality and stress coping: initial results from Bosnian young adults. J. Spiritual. Mental Health 18, 43–54. doi: 10.1080/19349637.2015.1059301

[ref29] Pew Research Center (2018). Eastern and western Europeans differ on importance of religion, views of minorities, and key social issues. https://www.pewforum.org/wp-content/uploads/sites/7/2018/10/Eastern-Western-Europe-FOR-WEB.pdf

[ref30] Räty L. K. A. Larsson B. M. W. Söderfeldt B. A. (2003). Health-related quality of life in youth: A comparison between adolescents and young adults with uncomplicated epilepsy and healthy controls. J. Adolesc. Health 33, 252–258. doi: 10.1016/S1054-139X(03)00101-0 14519566

[ref31] Saroglou V. (2002). Religion and the five factors of personality: A meta-analytic review. Personal. Individ. Differ. 32, 15–25. doi: 10.1016/S0191-8869(00)00233-6

[ref32] Saroglou V. (2021). The Psychology of Religion (The Psychology of Everything). London: Routledge.

[ref33] Saucier G. Skrzypińska K. (2006). Spiritual but not religious? Evidence for two independent dispositions. J. Pers. 74, 1257–1292. doi: 10.1111/j.1467-6494.2006.00409.x 16958702

[ref34] Sjöborg A. (2014). CRS-5 SWE in Heimbrock H.-G. Wyller T. Wolfteich C. Streib H. Breemer R. Van Den Casanova J. (Eds.), Secular and Sacred?: The Scandinavian Case of Religion in Human Rights, Law and Public space. Vandenhoeck & Ruprecht 236–260.

[ref35] Stefa-Missagli S. Huber H. P. Fink A. Sarlo M. Unterrainer H. F. (2014). Dimensions of religious/spiritual well-being, personality, and mental health: initial results from Italian college students. Arch. Psychol. Relig. 36, 368–385. doi: 10.1163/15736121-12341290

[ref36] Unterrainer H.-F. Huber H.-P. Ladenhauf K. H. Wallner-Liebmann S. J. Liebmann P. M. (2010a). MI-RSB 48. Diagnostica (Göttingen) 56, 82–93. doi: 10.1026/0012-1924/a000001

[ref37] Unterrainer H.-F. Ladenhauf K. H. Moazedi M. L. Wallner-Liebmann S. J. Fink A. (2010b). Dimensions of religious/spiritual well-being and their relation to personality and psychological well-being. Personal. Individ. Differ. 49, 192–197. doi: 10.1016/j.paid.2010.03.032

[ref38] Unterrainer H.-F. Ladenhauf K. Wallner-Liebmann S. Fink A. (2011). Different types of religious/spiritual well-being in relation to personality and subjective well-being. Int. J. Psychol. Relig. 21, 115–126. doi: 10.1080/10508619.2011.557003

[ref001] Unterrainer H.-F. Lewis A. J. Collicutt J. Fink A. (2013). Religious/spiritual well-being, coping styles, and personality dimensions in people with substance use disorders. International Journal for the Psychology of Religion 23, 204–213. doi: 10.1080/10508619.2012.714999

[ref39] Unterrainer H.-F. Lewis A. J. Fink A. (2014). Religious/spiritual well-being, personality and mental health: A review of results and conceptual issues. J. Relig. Health 53, 382–392. doi: 10.1007/s10943-012-9642-5, PMID: 22965652

[ref40] Unterrainer H.-F. Nelson O. Collicutt J. Fink A. (2012). The English version of the multidimensional inventory for religious/spiritual well-being (MI-RSWB-E): first results from British college students. Religions 3, 588–599. doi: 10.3390/rel3030588

[ref41] Van der Kloot W. Spaans A. Heiser W. (2005). Instability of hierarchical cluster analysis due to input order of the data. Psychol. Methods 10, 468–476. doi: 10.1037/1082-989X.10.4.468, PMID: 16393000

[ref42] Van Tilburg W. A. P. Igou E. R. Maher P. J. Moynihan A. B. Martin D. G. (2019). Bored like hell: religiosity reduces boredom and tempers the quest for meaning. Emotion, 19, 255–269. doi: 10.1037/Emo0000439, PMID: 29697990

[ref43] Wenzl M. Fuchshuber J. Podolin-Danner N. Silani G. Unterrainer H.-F. (2021). The Swedish version of the multidimensional inventory for religious/spiritual well-being: first results from Swedish students. Front. Psychol. 12:5191. doi: 10.3389/fpsyg.2021.783761, PMID: 34858301 PMC8630782

[ref44] Yonker J. Schnabelrauch C. DeHaan L. (2012). The relationship between spirituality and religiosity on psychological outcomes in adolescents and emerging adults: A meta-analytic review. J. Adolesc. 35, 299–314. doi: 10.1016/j.adolescence.2011.08.010, PMID: 21920596

